# Somatically Hypermutated *Plasmodium-*Specific IgM^+^ Memory B Cells Are Rapid, Plastic, Early Responders upon Malaria Rechallenge

**DOI:** 10.1016/j.immuni.2016.06.014

**Published:** 2016-08-16

**Authors:** Akshay T. Krishnamurty, Christopher D. Thouvenel, Silvia Portugal, Gladys J. Keitany, Karen S. Kim, Anthony Holder, Peter D. Crompton, David J. Rawlings, Marion Pepper

**Affiliations:** 1 Department of Immunology, University of Washington School of Medicine, Seattle, WA 98109, USA; 2 Center for Immunity and Immunotherapies, Seattle Children’s Research Institute, Seattle, WA 98101, USA; 3 Laboratory of Immunogenetics, National Institute of Allergy and Infectious Diseases, National Institutes of Health, Rockville, MD 20892, USA; 4 The Francis Crick Institute, Mill Hill Laboratory, The Ridgeway, Mill Hill, London NW7 1AA, UK

## Abstract

Humoral immunity consists of pre-existing antibodies expressed by long-lived plasma cells and rapidly reactive memory B cells (MBC). Recent studies of MBC development and function after protein immunization have uncovered significant MBC heterogeneity. To clarify functional roles for distinct MBC subsets during malaria infection, we generated tetramers that identify *Plasmodium*-specific MBCs in both humans and mice. Long-lived murine *Plasmodium*-specific MBCs consisted of three populations: somatically hypermutated immunoglobulin M^+^ (IgM^+^) and IgG^+^ MBC subsets and an unmutated IgD^+^ MBC population. Rechallenge experiments revealed that high affinity, somatically hypermutated *Plasmodium*-specific IgM^+^ MBCs proliferated and gave rise to antibody-secreting cells that dominated the early secondary response to parasite rechallenge. IgM^+^ MBCs also gave rise to T cell-dependent IgM^+^ and IgG^+^B220^+^CD138^+^ plasmablasts or T cell-independent B220^−^CD138^+^ IgM^+^ plasma cells. Thus, even in competition with IgG^+^ MBCs, IgM^+^ MBCs are rapid, plastic, early responders to a secondary *Plasmodium* rechallenge and should be targeted by vaccine strategies.

## Highlights

• Tetramers allow analyses of endogenous *Plasmodium*-specific B cells in mice and humans
• Three phenotypically distinct MBC populations form after murine malaria infection
• Plasmodium-specific IgM^+^ MBCs are somatically hypermutated and high affinity
• Plastic IgM^+^ memory B cells dominate the early response to malaria rechallenge

## Graphical Abstract

**Figure:**
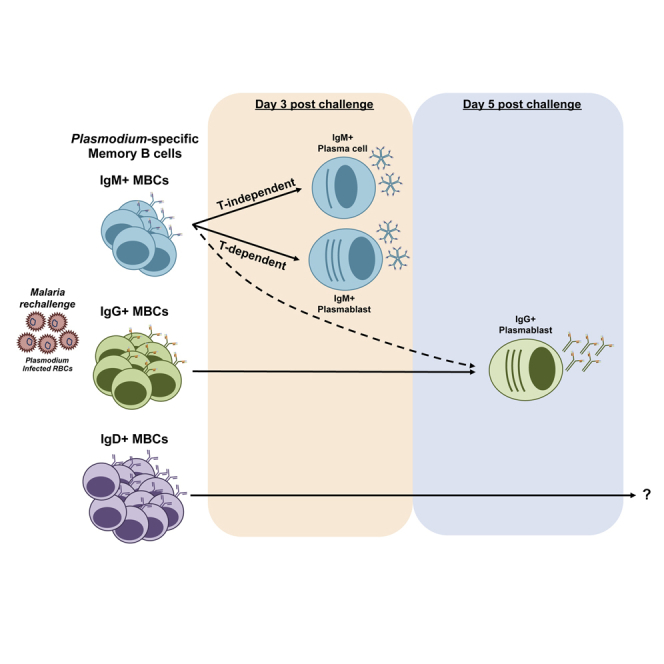

## Teaser

Heterogeneous types of memory B cells are present in both humans and mice, yet it is unclear how different MBC subsets form or function in response to infection. Pepper and colleagues reveal that phenotypically and functionally distinct populations of polyclonal *Plasmodium-*specific MBCs form in response to infection and somatically hypermutated, high-affinity, plastic IgM^+^ memory B cells dominate the early memory response to malaria rechallenge.

## Introduction

Memory B cells (MBCs) induced by vaccine or infection are critical components of a protective humoral response. MBCs can persist for long periods of time and rapidly respond to subsequent infection through the production of antibody-secreting cells, formation of new germinal centers (GCs), and repopulation of the memory pool (Tarlinton and Good-Jacobson, 2013). Classically defined MBCs express class-switched, somatically hypermutated B cell receptors (BCRs) after undergoing a GC reaction. These cells produce high-affinity antibodies within days of a secondary challenge, making them the gold standard for vaccine development. Recently, this homogeneous view of MBCs has been challenged and it is now recognized that diverse MBC subsets exist in both mice and humans (Dogan et al., 2009, Klein et al., 1997, Obukhanych and Nussenzweig, 2006, Pape et al., 2011, Seifert et al., 2015). Given this, it is critical for vaccine development to understand how distinct MBC populations respond to infection.

Technical advances in tracking antigen-specific B cells have revealed that MBCs are heterogeneous. They have been shown to express either isotype switched or unswitched BCRs that have undergone various degrees of somatic hypermutation (Kaji et al., 2012, Pape et al., 2011, Toyama et al., 2002). MBC subsets also exhibit varied expression of surface markers associated with T cell interactions such as CD73, CD80, and PDL2, revealing varied developmental histories and receptor ligand interactions (Anderson et al., 2007, Taylor et al., 2012b, Tomayko et al., 2010). Importantly, these phenotypically different MBC subsets have also been associated with functional heterogeneity, although different studies have led to different conclusions. Some studies have demonstrated that unswitched MBCs preferentially enter GCs while switched MBCs preferentially form plasmablasts (Benson et al., 2009, Dogan et al., 2009, Pape et al., 2011, Seifert et al., 2015). Other studies have shown instead that unswitched MBCs rapidly generate plasmablasts upon secondary challenge whereas switched MBCs preferentially re-enter GCs (McHeyzer-Williams et al., 2015). These are important distinctions to consider since different infections may have different requirements for humoral protection. Furthermore, the majority of these studies depended upon adoptive transfer of individual MBC subsets and/or were performed in models of protein immunization or after in vitro rechallenge. It therefore remains unclear how endogenous MBC subsets respond in competition during a secondary infection.

B cells play a critical role in immune protection to the blood stage of *Plasmodium* infection. The protective role for antibody was first demonstrated via passive transfer of hyperimmune immunoglobulin from adults to parasitemic children (Cohen et al., 1961), resulting in a dramatic decrease in blood stage parasitemia. Little is known, however, about the cellular source of *Plasmodium*-specific antibodies largely due to a lack of tools to analyze *Plasmodium*-specific B cells. We therefore generated B cell tetramers specific for the blood stage *Plasmodium* antigen, Merozoite Surface Protein 1 (MSP1). MSP1 is a key surface protein expressed by the parasite and is required for erythrocyte invasion (Kadekoppala and Holder, 2010). Antibodies generated against the 19kD C terminus region of MSP1 potently inhibit erythrocyte invasion and animals actively, or passively, immunized against MSP1 are protected against subsequent infection (Blackman et al., 1990, Hirunpetcharat et al., 1997, Moss et al., 2012). Furthermore, the acquisition of both IgG and IgM antibodies against the MSP1 C terminus have been associated with the development of clinical immunity (al-Yaman et al., 1996, Arama et al., 2015, Branch et al., 1998, Dodoo et al., 2008, Riley et al., 1992).

Tetramer enrichment techniques enabled the direct ex vivo visualization of rare *Plasmodium*-specific MBCs in malaria-infected humans and mice. We then performed detailed analyses of MSP1^+^ MBC formation and function in the rodent model of malaria, *Plasmodium chabaudi*. Both isotype-switched and unswitched MBCs emerged early in infection and persisted for at least 1 year. MSP1^+^ MBCs consisted of three distinct subsets including: classically defined, somatically hypermutated, high-affinity IgG^+^ MBCs, an IgM^low^IgD^high^ population that resembled naive B cells, and a third IgM^high^IgD^low^ MBC population that expressed somatically hypermutated BCRs that exhibit equivalent affinity to their IgG^+^ MBC counterparts. In response to various doses of malaria rechallenge, the majority of newly formed antibody-secreting cells (ASCs) were somatically hypermutated IgM^+^ cells, despite IgM^+^ MBCs being at a numerical disadvantage at the time of challenge. Furthermore, IgM^+^ MBCs produced both IgM and IgG antibody in response to rechallenge, thereby also contributing to the IgG^+^ antibody response 2 days later. Collectively, these studies demonstrate that *Plasmodium*-specific IgM^+^ MBCs are high-affinity, pluripotent early responders to malaria rechallenge that might provide a critical stop gap until IgG antibodies are generated and should therefore be considered in vaccine strategies.

## Results

### MSP1-Specific B Cells Expand, Differentiate, and Form Memory in Response to Blood Stage Malaria Infection

The direct ex vivo visualization of antigen-specific B cells during infection has been difficult to accomplish due to a lack of tools and techniques to track small population of B cells. We therefore adopted techniques used to analyze MBC development in response to protein immunization to study MBC development and function in response to blood stage malaria infection in C57BL/6 mice. To accomplish this, we generated a phycoerythrin (PE)-conjugated B cell tetramer containing the majority of the 19kD C-terminal portion of the MSP1 protein from *P. chabaudi* (Taylor et al., 2012a). This reagent was used with magnetic bead-based enrichment to analyze malaria-specific B cells directly ex vivo throughout all phases of the immune response.

**Figure 1:**
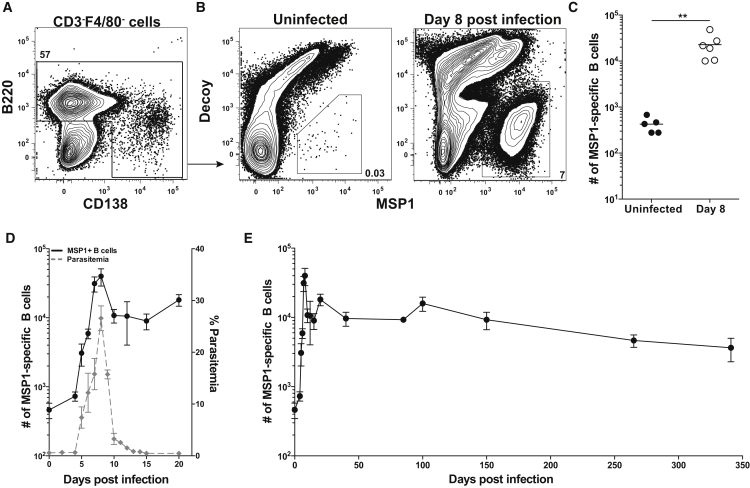
Detection and Kinetics of MSP1^+^ B Cells (A) Splenic B cells identified after excluding CD3^+^F4/80^+^ non-B cells and enrichment with MSP1 and Decoy tetramers. (B) Representative plots show MSP1^+^ B cells from (left) uninfected mice or (right) mice 8 days post-infection (p.i.). (C) Total number of MSP1^+^ B cells from uninfected or 8 days p.i. mice. Data are combined from two indpendent experiments with 5 or 6 mice per group. Line indicates mean ^∗∗^p < 0.01. (D) Kinetics of MSP1^+^ B cells (left y axis) and percent parasitemia (right y axis) over 20 days (E) Total MSP1^+^ B cells over 340 days p.i. For (D) and (E), each data point shows mean ± SEM with 3–8 mice per time point from at least two independent experiments. See also Figures S1 and S2.

In all experiments, splenocytes were first stained with a decoy reagent and then with the MSP1 PE tetramer to exclude cells binding other components of the tetramer (Taylor et al., 2012a). Anti-PE coated magnetic beads were then used to enrich both decoy-specific and MSP1-specific B cells, which were subsequently stained with antibodies for analysis by multiparameter flow ctometry. Antibody panels were based upon gating strategies developed to visualize all stages of mature B2 B cell differentiation. After excluding non-lymphocytes and doublets, Decoy^−^MSP1^+^ B cells were identified among B220^+^ and B220^low^CD138^+^ cells (identifying plasmablasts) (Figures 1A and 1B). In uninfected mice, there were approximately 400 MSP1^+^ B cells, while 8 days after infection with 1 × 10^6^ *P. chabaudi* iRBCs (Butler et al., 2012), the number of MSP1^+^ B cells expanded 50-fold to 23,000 cells (Figures 1B and 1C). Control experiments demonstrated that B cells with BCRs specific for hen egg lysozyme (MD4 *Rag2*^−/−^ mice) did not bind the MSP1 tetramer nor were they activated non-specifically by *Plasmodium* 8 days post-infection after adoptive transfer into a congenic host (Figures S1A and S1B). Thus, rare endogenous MSP1^+^ B cells that could be identified in naive mice, expanded in an antigen-specific manner demonstrating our ability to stringently identify and analyze MSP1^+^ B cells throughout the course of *Plasmodium* infection.

Both parasitemia and MSP1^+^ B cells were quantified in the spleens of individual mice for approximately a year after infection. Parasitemia was measured in blood samples throughout the course of infection using a flow cytometry based assay (Malleret et al., 2011, Robbiani et al., 2015) (Figure S2A). MSP1^+^ B cells isolated from spleens of infected mice began to expand by 4 days after infection, peaked 8 days after infection, then sharply contracted, mirroring parasitemia (Figure 1D and Figure S2B). Variations in total MSP1^+^ B cell numbers continued until day 150 although intracellular staining with the cell-cycle marker Ki67 demonstrated that the vast majority (∼95%) of MSP1^+^ B cells at day 100 are quiescent (data not shown). MSP1^+^ B cells persisted with a half-life of 221 days that resulted in a population of 3,600 cells at 340 days post infection (Figure 1E). MSP1^+^ B cells therefore expanded with ascending parasitemia, contracted, and then numbers fluctuated before stabilizing and slowly declining over 350 days. Importantly, these data demonstrated that long-lived, quiescent *Plasmodium*-specific B cells persisted and could be analyzed well after parasitemia is controlled.

### MSP1-Specific B Cell Fates Emerge Early after Infection and MBCs Persist

**Figure 2:**
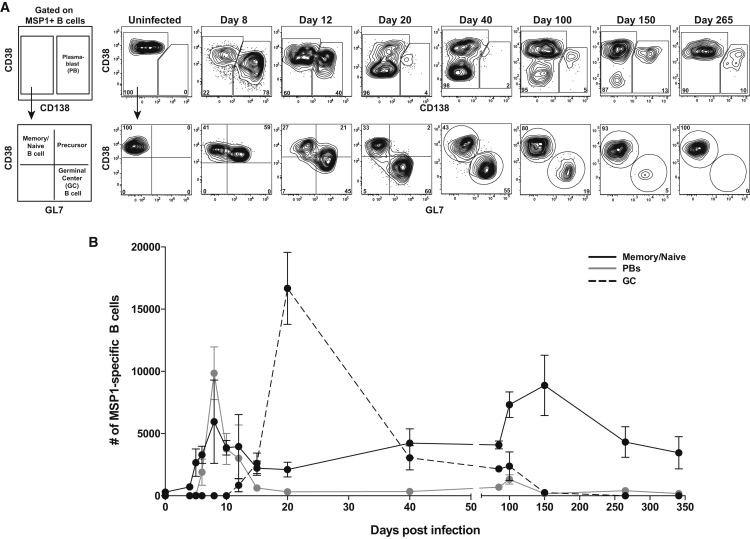
MSP1^+^ B Cell Fates Emerge Early after Infection and MBCs Persist (A) Gating scheme and representative plots of MSP1^+^ B cells. CD138^+^ cells (top row) and CD138^−^ memory/naive cells, precursor cells, and GC B cells (bottom row) over 265 days post infection. (B) Total MSP1^+^ MBCs/naive, plasmablasts, and GC B cells. Each data point shows mean ± SEM with 3–8 mice per time point from at least two independent experiments. See also Figure S3.

The heterogeneity of MSP1^+^ B cells was first assessed during the acute phase of the infection. Gating strategies were designed to distinguish between CD138^+^ plasmablasts (PBs), CD38^+^GL7^+^ activated precursors (Taylor et al., 2012b), CD38^−^GL7^+^ germinal center (GC) B cells, and expanded CD38^+^GL7^−^ MBC populations (Figure 2A). Within 8 days of infection, multiple fates emerged including a dominant population of MSP1^+^CD138^+^ PBs that primarily expressed IgM as measured by flow cytometry and serum ELISA consistent with previous reports (Achtman et al., 2003, Nduati et al., 2010) (Figures 2A, S3A, S3D). Several thousand MSP1^+^ B cells that retained CD38 expression, therefore resembling MBCs, were also present at day 8. The remainder of the population consisted of IgM^+^ and IgM^−^ GL7^+^CD38^+^ activated precursors, which have been shown to be multipotent and capable of differentiating into GC B cells or MBCs (Figures 2A and 2B, S3B) (Taylor et al., 2012b). While GC responses were not present at day 8, they began to emerge at day 12, and expanded to a peak of about 15,000 MSP1^+^GL7^+^IgM^−^IgD^−^CD38^−^ cells at day 20, at which point numerous IgD^−^ germinal centers could also be found in the spleen by immunofluorescent microscopy (Figures 2A and 2B, S3B, S3C). This was further confirmed by the presence of various sub-classes of MSP1-specific IgG^+^ antibodies measured in the serum (Figure S3D).

To determine which of these early fates persisted into the memory phase of the response, we characterized MSP1^+^ B cells for approximately a year using similar gating strategies described above (Figures 2A and 2B). Although CD138^+^ PBs initially waned betweens days 20 to 40, a small, consistently present CD138^+^ population re-emerged around day 85 suggesting that these were splenic plasma cells (PCs), similar to recent work demonstrating that PCs emerge after MBCs in response to protein immunization (Bortnick et al., 2012, Weisel et al., 2016). These PCs persisted at all time points thereafter, were still present at day 340 post infection, and were predominantly IgM^+^ (Figures 2A and 2B, S3A).

Enrichment techniques also facilitated the visualization of a waning GC response. MSP1^+^ GC B cells contracted by day 40 post infection and then slowly declined before eventually disappearing around 150 days post infection. Therefore, from day 50 on, the vast majority of the MSP1^+^ cells were CD38^+^GL7^−^ MBCs that remained for at least 340 days post infection (Figures 2A and 2B). These data demonstrate that well after parasite clearance and termination of the GC reaction, splenic MSP1^+^ B cells were composed of an expanded population of CD38^+^ MBCs and a small but persistent CD138^+^ PC population.

### Switched and Unswitched *Plasmodium*-Specific MBCs Can Be Found in Malaria-Exposed Mice and Humans

**Figure 3:**
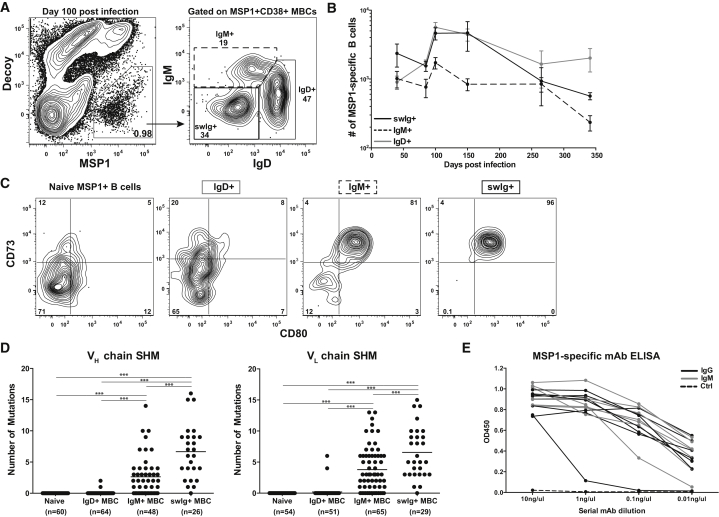
MSP1^+^ MBCs Are Heterogeneous (A) Representative plot of MSP1^+^ MBCs and isotype of MSP1^+^CD38^+^ MBCs to identify IgD^+^, IgM^+^, and swIg^+^ MBCs 100 days p.i. (B) Total number of MSP1^+^ IgD^+^, IgM^+^, and swIg^+^ MBCs from day 40 to 340 p.i. Each data point shows mean ± SEM with 3–8 mice per time point from at least two independent experiments. (C) Representative plots of CD73 and CD80 on MSP1^+^ naive B cells or IgD^+^, IgM^+^, and swIg^+^ MBCs 100 days p.i. (D) Number of mutations in the heavy chain (V_H_) or light chain (V_L_) of individual MSP1^+^ naive B cells or CD73^−^CD80^−^IgD^+^, CD73^+^CD80^+^IgM^+^, or CD73^+^CD80^+^swIg^+^ MBCs 100 days p.i. Each dot indicates a single cell. Line indicates mean. Data combined from three independent experiments. ^∗∗∗^p < 0.001. (E) ELISA of serially diluted MSP1-specific IgM^+^ and swIg^+^ mAbs. Each line represents a single clone. OD_450_, optical density at 450 nm. See also Figure S4.

It was next important to determine whether recently defined MBC subsets that emerge after protein immunization were also present in response to infection. To interrogate the diversity of the MSP1^+^ MBCs, we used antibodies specific for IgM and IgD to identify “switched” and “unswitched” MSP1^+^ B cells. Interestingly, this staining strategy identified three distinct populations of MSP1^+^ MBCs 100 days after infection: an IgM^−^IgD^−^ isotype switched population (referred to as swIg^+^) and two unswitched subsets. One subset was phenotypically IgM^lo^IgD^high^ (referred to as IgD^+^) while the other subset was IgM^high^IgD^lo^ (referred to as IgM^+^) (Figure 3A). While all three populations persisted for 340 days post infection, at the latest time points IgD^+^ MBCs stably persisted, whereas both the IgM^+^ and swIg^+^ MBCs declined (Figure 3B).

The persistence of heterogeneous MBCs after malaria infection in mice led us to ask whether switched and unswitched *P. falciparum*-specific MBCs also occur in exposed individuals residing in an endemic area. Although IgG^+^ *P. falciparum*-specific MBCs have been detected by ELISPOT (Weiss et al., 2012) in individuals exposed to both high (Ndungu et al., 2012, Weiss et al., 2010) and low (Clark et al., 2012, Ndungu et al., 2013, Wipasa et al., 2010) malaria transmission, it is unknown whether *P. falciparum* infection induces antigen-specific unswitched MBCs. Antigen-specific enrichment experiments were therefore performed on peripheral blood mononuclear cells (PBMCs) collected from *P. falciparum*-infected Malian subjects during the malaria season (Crompton et al., 2008) or malaria-naive U.S. subjects. To enhance the sensitivity of *Plasmodium*-specific cell detection (less than 20 million PBMC were available in some samples), we generated B cell tetramers using the C-terminal region of MSP1 and apical membrane antigen 1 (AMA1) from the human *P. falciparum* (*3D7*) strain. In Malian subjects, we found that approximately 40% of the *P. falciparum*-specific B cells in blood were CD21^+^CD27^+^ MBCs in keeping with expected frequencies of total MBCs in human blood (Kaminski et al., 2012, Klein et al., 1998, Tangye and Good, 2007) (Figure S4A). Furthermore, there was a 6-fold increase in the total number of *P. falciparum*-specific B cells and a 60-fold increase among CD27^+^CD21^+^ MBCs compared to uninfected U.S. controls (Figure S4B). We further characterized the *P. falciparum*-specific CD27^+^ MBCs from Malian samples for their expression of BCR isotype and found that they comprised both switched and unswitched cells (Figure S4C). Thus, heterogeneous populations of *Plasmodium*-specific MBCs are expanded in both mice and humans and future studies will address similarities and differences between these populations.

### Murine MSP1-Specific MBC Subsets Are Phenotypically and Genetically Distinct

To further dissect the unique phenotypic and functional characterists associated with distinct *Plasmodium*-specific MBCs, we performed additional studies in mice. Previous studies have demonstrated that MBC subsets display heterogeneous expression of surface markers associated with T cell interactions including CD73 and CD80 on both switched and unswitched MBCs (Anderson et al., 2007, Tomayko et al., 2010, Yates et al., 2013). Expression of these proteins was therefore examined on MSP1^+^ MBCs 100 days post infection. Again we found that the division of unswitched MBCs into IgM^+^ and IgD^+^ subsets largely accounted for the variability in surface marker expression. ∼81% of IgM^+^ MBCs expressed CD73 and CD80 comparable to the ∼96% of the swIg^+^ MBCs that expressed both markers, whereas only ∼8% of IgD^+^ MBCs expressed CD73 and CD80, comparable to MSP1^+^ naive B cells (Figure 3C). Similar to MBC diversity generated by protein immunization, phenotypically diverse *Plasmodium*-specific IgD^+^, IgM^+^, and swIg^+^ MBC subsets develop in response to infection. Additionally, expression of CD73 and CD80 further distinguishes IgM^+^ and IgD^+^ MBCs as two distinct, unswitched populations.

B cell expression of both CD73 and CD80 is associated with expression of activation-induced cytidine deaminase (AID) and in some cases but not all, germinal center dependence (Anderson et al., 2007, Kaji et al., 2012, Taylor et al., 2012b, Weisel et al., 2016). On the basis of these observations, we hypothesized that our CD73^+^CD80^+^MSP1^+^IgM^+^ MBCs might represent a previously unexplained population of somatically hypermutated, unswitched MBCs identified in other immunization models (Kaji et al., 2012, Pape et al., 2011). To test this hypothesis, we used flow cytometric sorting to isolate individual MSP1^+^ CD73^−^CD80^−^IgD^+^, CD73^+^CD80^+^IgM^+^, and CD73^+^CD80^+^swIg^+^ MBCs or MSP1^+^ naive B cells. We sequenced and cloned individual BCRs using previously described methods (Tiller et al., 2009). The relative numbers of somatic hypermutations (SHM) in both heavy (V_H_) and light (V_L_) chain sequences present in individual MBC subsets were calculated after comparison to BCRs from naive MSP1^+^ B cells, which had no mutations and were identical to germline sequences. While only 3% of IgD^+^ MBC V_H_ or V_L_ chain sequences showed SHM, 65% of V_H_ and 75% of V_L_ chain sequences of CD73^+^CD80^+^IgM^+^ cells were mutated with a mean of 3 mutations in both chains (Figure 3D). As expected, swIg^+^ MBCs were also highly mutated (97%) and displayed significantly more mutations (mean of 8) in both V_H_ and V_L_ chains (Figure 3D).

Because increased levels of SHM are associated with an overall increase in BCR affinity (Chan and Brink, 2012), we tested the affinities of IgM^+^ and swIg^+^ MBC BCRs. Individual BCR variable region sequences with varying levels of somatic hypermuation from either MSP1^+^ IgM^+^ or swIg^+^ MBC clones were therefore expressed as monoclonal antibodies (mAb) with human IgG constant (Fc) regions to prevent contributions to avidity by oligomerization. Antibodies were then used in dilution assays against MSP1 protein to compare affinity of the various mAbs by ELISA (Kolhatkar et al., 2015). Importantly, these studies further confirm the specificity of our MSP1-tetramer techniques as 100% of expressed clones bound MSP1 protein, whereas the control PC-specific mAb did not (Figure 3E). Furthermore, despite overall fewer mutations, individual BCRs from IgM^+^ MBCs showed comparable affinity for the MSP1 protein to swIg^+^ MBCs (Figure 3E). These data therefore demonstrate that expression of CD73 and CD80 on both IgM^+^ and swIg^+^ MBCs is associated with increased levels of SHM, resulting in similar BCR affinities. These findings raise the question of how these MBCs might respond in competition during a secondary infection.

### Secondary Infection Induces the Rapid Proliferation and Differentiation of MSP1-Specific MBCs

**Figure 4:**
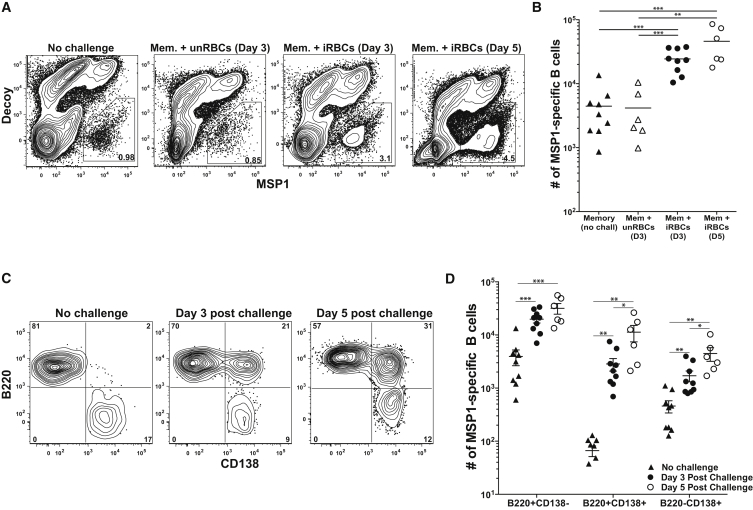
Rapid Expansion of MSP1^+^ B Cells after Rechallenge (A) Representative plots identifying MSP1^+^ B cells in memory mice rechallenged with 1 × 10^7^ unRBCs or iRBCs and analyzed 3 or 5 days later. (B) Total number of MSP1^+^ B cells in (A). Data combined from two independent experiments with 6–9 mice per group. Line indicates mean. ^∗∗^p < 0.01,^∗∗∗^p < 0.001. (C) Representative plots of B220 by CD138 on MSP1^+^ B cells in memory mice rechallenged with 1 × 10^7^ iRBCs analyzed 3 or 5 days later. (D) Total number of MSP1^+^ B220^+^CD138^−^, B220^−^CD138^+^, and B220^+^CD138^+^ cells in C. Data combined from two independent experiments with 6–9 mice per group. Line indicates mean. ^∗^p < 0.05, ^∗∗^p < 0.01,^∗∗∗^p < 0.001. See also Figure S5.

To understand how the MBCs described above function during a secondary infection, we rechallenged mice in the memory phase of the response with iRBCs. Of note, our experimental conditions were distinct from several previous studies that utilized adoptive transfer of individual MBC populations followed by antigen rechallenge. In intact memory mice, MBC competition for antigen and T cell help, as well as the presence of pre-existing antibodies factor into the overall response, perhaps as they would in repeatedly infected humans. To accomplish this, memory mice infected 12–16 weeks prior were left unchallenged or rechallenged with either 1 × 10^7^ uninfected RBCs (unRBCs) or iRBCs and MSP1^+^ B cells were analyzed 3 or 5 days later. Following rechallenge with iRBCs, but not unRBCs, the total number of MSP1^+^ B cells expanded significantly on day 3 and continued to increase at day 5 compared to unchallenged memory mice (Figures 4A and 4B). To ascertain whether these newly formed cells were originating from MBCs or recently formed naive cells, we also infected naive mice with a challenge dose of 1 × 10^7^ iRBCs and quantified and phenotyped MSP1^+^ B cells. In stark contrast to the logarithmic increase seen in MSP1^+^ B cells in memory mice after rechallenge, there was no significant increase in the total number of MSP1^+^ B cells in naive mice at either 3 or 5 days after a primary infection (Figure S5A).

We next to determined whether expanded MSP1^+^ cells in rechallenged memory mice were also differentiated. Phenotypic analyses using gating strategies described above confirmed that MSP1^+^ B cells in memory mice prior to challenge consisted of both B220^+^CD138^−^ B cells (consisting primarily of MBCs and a small, waning population of GC B cells) and B220^−^CD138^+^ PCs (Figures 2 and 4C). Three days after iRBC challenge, a newly formed MSP1^+^B220^+^CD138^+^ population emerged and remained expanded at day 5, suggesting these were the product of recently activated MBCs (Figures 4C and 4D). The rapid formation of this population was unique to a memory response as we did not observe a significant B220^+^CD138^+^ population form in naive mice 3 days after the same iRBC challenge (Figure S5B). Additional quantification of the B220^+^CD138^−^ B cells and B220^−^CD138^+^ PCs revealed that these populations also increased in number after rechallenge (Figure 4D). Together, these data demonstrate that within 3 days of rechallenge, expanded and differentiated MSP1^+^ B cells form in response to a secondary infection.

To determine what precursor populations were proliferating to produce expanded populations of MSP1^+^ B cells, we compared Ki67 expression (which marks actively cycling cells) before and after rechallenge. Prior to challenge, ∼4% of MSP1^+^ B cells were Ki67^+^ (Figure S6A). Three days after rechallenge, the percentage of Ki67^+^ increased to ∼16% of all MSP1^+^ B cells and remained restricted to the B220^+^ B cells (B220^−^ PCs were Ki67^−^) (Figure S6A). Detailed phenotypic analysis of the Ki67^+^ cells revealed that three separate MSP1^+^B220^+^ populations were proliferating: newly formed B220^+^CD138^+^ plasmablasts (PBs) (∼30%), CD38^+^ MBCs (∼50%), and CD38^+^GL7^+^ activated precursors (∼20%) (Figure S6A). Therefore, within 3 days, some MSP1^+^ MBCs had already proliferated and differentiated into PBs and CD38^+^GL7^+^ activated precursors, but many CD38^+^GL7^−^ MBCs were still proliferating but had not yet differentiated.

The isotypes of the proliferating cells were also determined to reveal precursor relationships. Surprisingly, the majority of both Ki67^+^ PBs 3 days after rechallenge expressed IgM despite IgM^+^ MBCs being at a numerical disadvantage to the swIg^+^ MBCs at this time point (Figure S6B, Figure 3B). The activated precursors and MBCs were largely isotype switched (Figure S6B). In contrast, very few of the MSP1^+^ IgD^+^ MBCs were proliferating. These data demonstrate that IgM^+^ MBCs rapidly respond to secondary infection and make up the majority of the early proliferating plasmablasts.

To further discern precursor relationships for the IgM^+^ PBs, we cloned BCRs from the IgM^+^B220^+^CD138^+^ PBs 3 days after challenge to look for somatic hypermutation. If the PBs were somatically hypermutated, it would support the idea that these cells were derived from somatically hypermutated IgM^+^ MBCs as opposed to unmutated IgD^+^ MBCs. Remarkably, 95% of newly formed IgM^+^ PB clones (mean mutation of 8) were somatically hypermutated at levels that were comparable to MSP1^+^IgM^+^ MBCs, further establishing a precursor relationship between IgM^+^ MBCs and newly formed PBs after a secondary infection (Figure S6C).

### The Early Secondary Antibody Response is IgM-Dominant

**Figure 5:**
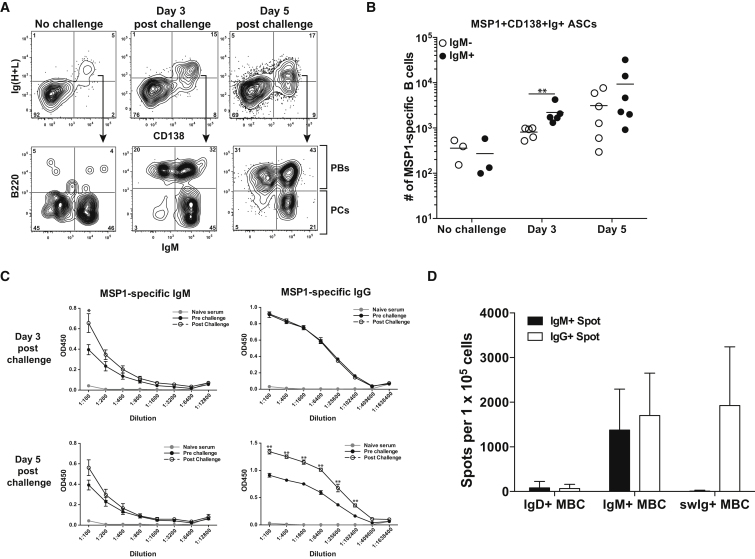
Early Secondary Antibody Response Is IgM-Dominant (A) Representative plots of intracellular Ig(H+L) (Ig) and CD138 expression of MSP1^+^ B cells. Bottom row shows B220 by IgM expression of MSP1^+^Ig^+^CD138^+^ cells in memory mice pre challenge or 3 or 5 days post challenge with 1 × 10^7^ iRBCs. (B) Total number of all IgM^+^ and IgM^−^ MSP1^+^ CD138^+^Ig^+^ cells in (A). Data combined from two independent experiments with 3–6 mice per group. Line indicates mean. ^∗∗^p < 0.01. (C) MSP1-19 IgM and IgG ELISA from serum of individual memory mice prechallenge and 3 or 5 days post challenge. OD_450_, optical density at 450 nm. Each dilution point shows mean ± SEM. Graphs represent combined data from three independent experiments with three mice per group. ^∗^p < 0.05, ^∗∗^p < 0.01. (D) ELISPOT on MSP1^+^ IgD^+^, IgM^+^, and swIg^+^ MBCs sorted from memory mice 2 days post challenge. Data compiled from five mice in two independent experiments. Error bars show SD. See also Figure S6.

We next asked what MSP1^+^ cells were differentiated antibody secreting cells (ASCs). Again, memory mice were rechallenged and intracellular staining for immunoglobulin heavy and light chain (Ig) was performed on MSP1^+^ B cells 3 or 5 days later. In memory mice analyzed prior to challenge, the only ASCs present were ∼600 B220^−^CD138^+^ PCs (which represent about 5% of the total cells) (Figure 5A). Three days after rechallenge, approximately **∼**3,000 MSP1^+^ B cells (about 15%) were now making antibody, split between B220^+^CD138^+^ PBs and B220^−^CD138^+^ PCs (Figure 5A). Now, approximately 70% of the MSP1^+^Ig^+^ ASCs were IgM^+^, while only about 30% of the ASCs were switched, resulting in significantly more IgM^+^ ASCs on day 3 than switched ASCs (Figures 5A and 5B). Two days later, on day 5 post challenge, IgM^+^ ASCs continued to expand, but now there was also a larger, switched antibody-secreting PB pool. Interestingly, the switched PCs stayed relatively stable at all time points examined (Figures 5A and 5B).

To confirm that our intracellular antibody staining represented measurable changes of secreted antibody in vivo, we performed MSP1-19 protein-specific ELISAs on serum samples taken from individual mice before or after challenge. In conjunction with what was observed by flow cytometry, 3 days after infection MSP1-specific IgM antibody expression was significantly increased over pre-challenge levels while IgG antibody expression remained unchanged (Figure 5C, top row). Two days later however, on day 5, we observed significant increases in MSP1-specific IgG antibodies, while IgM antibody levels remained elevated (Figure 5C, bottom row). Because it was unclear whether these switched PBs arose from swIg^+^ or IgM^+^ MBCs, we additionally sorted MBCs 2 days after rechallenge to look for IgM or IgG expression by ELISPOT after 2 days in culture. This approach revealed that whereas swIg^+^ MBCs could only form IgG^+^ ASCs, IgM^+^ MBCs formed both IgM^+^ and IgG^+^ ASCs (Figure 5D). Collectively, these data demonstrate that the secondary response is dominated by early IgM^+^ antibody expression and later IgG^+^ antibody expression. Additionally, our findings demonstrate that IgM^+^ MBCs are capable of expressing both IgM^+^ and IgG^+^ antibodies, highlighting that the IgM^+^ MBCs are rapid, plastic responders to a secondary infection.

### Secondary IgM Response Is Not Affected by Challenge Dose or Timing

**Figure 6:**
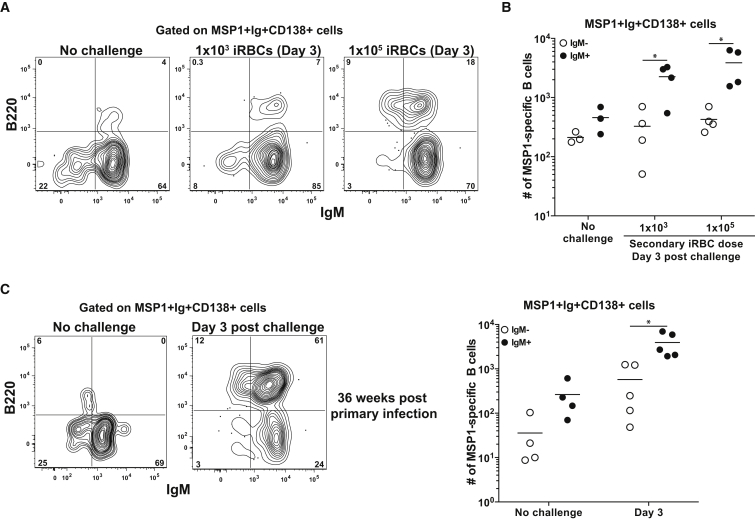
iRBC Challenge Dose or Timing Does Not Impact Secondary IgM Response (A) Representative plots of B220 and IgM expression on MSP1^+^Ig^+^CD138^+^ cells in memory mice (12–16 weeks post primary infection) pre challenge or 3 days post challenge with 1 × 10^3^ or 1 × 10^5^. (B) Total number of all IgM^+^ and IgM^−^ MSP1^+^ CD138^+^Ig^+^ cells in A. Data combined from two independent experiments with 3 or 4 mice per group. Line indicates mean. ^∗^p < 0.05. (C) Representative plots of B220 and IgM expression on MSP1^+^Ig^+^CD138^+^ cells (left) and total number of all IgM^+^ and IgM^−^ MSP1^+^ CD138^+^Ig^+^ cells (right) in memory mice 36 weeks p.i. prior to challenge or 3 days post challenge with 1 × 10^7^ iRBCs. Data combined from two independent experiments with 4 or 5 mice per group. Line indicates mean. ^∗^p < 0.05

One potential cause for the early IgM dominant response after secondary challenge could be a high antigen load, which could somehow preferentially activate IgM^+^ MBCs. Memory mice were therefore challenged with two lower iRBC challenge doses (1 × 10^3^ and 1 × 10^5^) prior to MSP1^+^ B cell analysis 3 days later. Remarkably, in both lower dose challenges, the IgM^+^ ASC response still dominated the early ASC population and even more dramatically than what we had observed at the higher dose challenge (Figures 6A, 6B, and 5B). This was especially striking given the 2.5-fold numerical disadvantage of IgM^+^ MBCs compared to swIg^+^ MBCs 100 days post-challenge (Figure 3B).

Although this ruled out dose dependent effects, it was also possible that the time of rechallenge influenced our results, for example if a germinal center was ongoing, which was the case for the 12–16 week rechallenge experiments. We therefore tested whether the presence of an ongoing GC reaction at the time of challenge influenced the early secondary responders. Memory mice 35 weeks post infection, in which the GC reaction had ended and IgM^+^ and swIg^+^ MBCs were in equal number (Figures 2 and 3), were therefore given a secondary challenge with 1 × 10^7^ iRBCs and analyzed 3 days later. As seen in mice with an ongoing GC, IgM^+^ cells were still the predominant early antibody-expressing population (Figure 6C). Together, these data suggest that despite variations in infectious dose, the presence or absence of a GC, or shifts in the numerical ratio of IgM^+^ to swIg^+^ MBCs, IgM^+^ MBCs can compete with swIg^+^ MBCs and are important early responders in a secondary *Plasmodium* infection.

### IgM^+^ MBCs Generate Both T-Independent and T-Dependent Antibody Secreting Effectors

**Figure 7:**
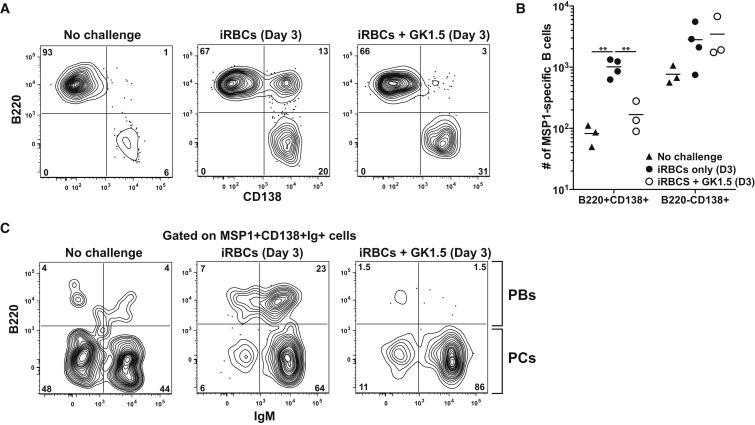
Requirements for Secondary IgM^+^ MBC Responses (A) Representative plots of MSP1^+^ B cell B220 and CD138 expression after iRBC rechallenge ± CD4 depletion (GK1.5). (B) Total number of MSP1^+^ B220^+^CD138^+^ cells and B220^-^CD138^+^ cells in (A). Data are combined from two independent experiments with 3-4 mice per group. Line indicates mean. ^∗∗^p < 0.01. (C) Representative plots of B220 and IgM expression on MSP1^+^Ig^+^CD138^+^ cells in (A).

The predominant secondary IgM^+^ memory response led us to interrogate the mechanisms of the early IgM response. We hypothesized that differences in T cell dependence could perhaps allow some populations to form faster than others. To test this, mice were treated a CD4^+^ T cell-depleting antibody (clone GK1.5) for 2 days prior to rechallenge and formation of PBs and PCs was assessed 3 days later. Strikingly, while MSP1^+^ PBs did not form in the absence of T cell help, the PCs in the GK1.5 treated animals expanded comparably to those in a T cell replete rechallenged mouse (Figures 7A and 7B). To assess the isotype of the responding T-independent ASCs, we again performed intracellular Ig staining. In mice depleted of T cells, more than 85% of the Ig^+^CD138^+^ ASCs expressed IgM^+^ (Figure 7C). Therefore, the formation of both unswitched and switched PBs is T cell dependent yet predominantly IgM^+^ expressing PCs can still form in a T cell independent manner. These data therefore suggest that IgM^+^ MBCs can form two unique ASC populations in two mechanistically distinct ways, again highlighting their plasticity.

## Discussion

Here we focused on understanding how recently described MBC subsets develop and function in response to infection with a relevant pathogen. To accomplish this, we generated B cell tetramers and utilized enrichment techniques to perform analyses of endogenous *Plasmodium*-specific B cells in malaria-exposed humans and mice. Importantly, the results presented in these studies highlight the fact that IgM^+^ and IgD^+^ MBCs are unique populations of cells with distinct phenotypic, functional, and survival properties. Furthermore, these studies emphasize that IgM^+^ MBCs are not low affinity cells that provide redundancy to IgG^+^ MBCs. On the contrary, *Plasmodium*-specific IgM^+^ MBCs express high affinity and somatically hypermutated BCRs and rapidly respond to produce antibodies prior to IgG^+^ MBCs, even in competition. Lastly, these studies reveal that a secondary memory response results in the generation of T-dependent plasmablasts and T-independent plasma cells that create multiple layers of antibody secreting cells.

In many ways, the results presented reconcile many of the disparate findings from various studies using a variety of protein immunization strategies, BCR transgenics, and isolated transfer and rechallenge techniques. Dividing unswitched cells into two populations based on differential expression of IgM and IgD revealed that IgM^+^ MBCs were far more similar in phenotype (CD73 and CD80 expression), developmental history (evidence of somatic hypermutation), affinity, survival, and function (rapid plamablast formation) to swIg^+^ MBCs than the more naive-like IgD^+^ MBCs. Thus either isotype, as shown by Pape et al. (Pape et al., 2011) or expression of markers associated with somatic hypermutation (Zuccarino-Catania et al., 2014) can predict MBC function, reconciling the findings of these two separate studies. While the IgD^+^ MBCs were remarkably stable, both the IgM^+^ and swIg^+^ subsets persisted with similar, and less stable kinetics as predicted by studies demonstrating a loss of somatically hypermutated B cells over time (Gitlin et al., 2016). IgD^+^ MBCs might represent a durable, expanded memory population that provides a high number of pathogen-specific clones with kinetics similar to naive B cells.

We have also addressed how distinct antigen-specific MBC subsets respond to a secondary infection in vivo in competition and demonstrate a hierarchy of MBC responsiveness to secondary infection. Surprisingly, at the earliest time points, IgM^+^ MBCs are the dominant producers of ASCs at all doses of rechallenge and time points examined. By 5 days post secondary infection, however, IgM antibody production did not continue to increase while switched PBs began to produce significant amounts of antibody highlighting that this dominance is transient. Therefore, unlike previous studies suggesting that IgM cells do not readily form PBs perhaps due to their low affinity (Pape et al., 2011) or form plasmablasts with similar kinetics to IgG^+^ PBs (Zuccarino-Catania et al., 2014), in this system, IgM^+^ MBCs are high affinity, rapid, plastic early responders that appear to initate the secondary response.

Our results might explain recent data associating the depth and breadth of *Plasmodium*-specific IgM antibodies with resistance to infection (Arama et al., 2015). While we demonstrate that F(ab)s made from IgM^+^ MBCs are of comparable affinity to those sequenced from IgG^+^ MBCs, upon pentamerization of IgM antibodies, the IgM^+^ antibody avidity would be far greater than the IgG antibodies. Moreover, IgM antibodies are important mediators of complement mediated lysis, which is important for control of blood stage infection (Boyle et al., 2015). While the importance of IgM antibodies in *Plasmodium* infection has been shown in murine models (Couper et al., 2005), additional studies examining the importance of IgM antibodies in human malaria infection as well as the comparison of *Plasmodium*-specific IgM^+^ MBCs found in our murine system to those we identified in malaria-exposed humans are necessary and ongoing.

Finally, these studies help to clarify long-standing controversies concerning the level of T cell dependence of secondary MBC responses (Kurosaki et al., 2015). Although many studies in humans and mice have demonstrated T cell-independent activation of MBCs (Bernasconi et al., 2002, Richard et al., 2008, Von Eschen and Rudbach, 1974), later studies suggested MBCs cannot be activated by bystander inflammation (Benson et al., 2009) or without the help of T cells (Ise et al., 2014). Our results demonstrate that both T-dependent and T-independent processes contribute to a secondary MBC response and support recent studies demonstrating that IgM^+^ MBCs can be reactivated in a T-independent manner when transferred in isolation into T cell-depleted mice (Zuccarino-Catania et al., 2014). Specifically, secondary IgM^+^ and IgG^+^ PB formation was T-dependent, while the rapid generation of non-dividing, antibody-secreting IgM^+^ PCs was T-independent, raising many questions about the origins of these cells. It is tempting to speculate that the murine somatically hypermutated IgM^+^ MBCs identified in these studies are homologous to human IgM^+^ MBCs that can mediate T-independent IgM^+^ responses to bacterial infection (Weill et al., 2009). In conclusion, these studies highlight the IgM^+^ MBC as a functional, plastic, rapidly responding MBC population that should be targeted by vaccines to prevent disease.

## Experimental Procedures

### Animals

5- to 8-week-old female C57BL/6 and B6.SJL-Ptprc^a^ Pepc^b^/BoyJ (CD45.1^+^) mice were used for these experiments. Mice were purchased from The Jackson Laboratory and maintained/bred under specific pathogen free conditions at the University of Washington. MD4-*Rag2*^−/−^ mice were provided by Dr. Marc Jenkins (University of Minnesota). All experiments were performed in accordance with the University of Washington Institutional Care and Use Committee guidelines.

### Plasmodium Infection

*Plasmodium chabaudi chabaudi (AS)* parasites were maintained as frozen blood stocks and passaged through donor mice. Primary mouse infections were initiated by intraperitoneal (i.p.) injection of 1 × 10^6^ iRBCs from donor mice. Secondary mouse infections were performed 12–35 weeks after primary infection using a dose of 1 × 10^7^ iRBCs injected intravenously (i.v.). In some cases, when indicated, secondary challenges were given at lower doses using either 1 × 10^3^ or 1 × 10^5^ iRBCs injected i.v.

### Tetramer Production

For murine studies, recombinant His-tagged C-terminal MSP1 protein (amino acids 4960 to 5301) from *P. chabaudi (AS)* (provided by Dr. Jean Langhorne, Francis Crick Institute) was produced by *Pichia pastoris* and purified using a Ni-NTA agarose column as previously described (Ndungu et al., 2009). Purified *P. chabaudi* MSP1 protein was biotinylated and tetramerized with streptavidin-PE (Prozyme) as previously described (Taylor et al., 2012a). For human studies, AMA1 protein from *P. falciparum (3D7)* (provided by Dr. Julian Rayner, Welcome Trust Sanger Institute) and MSP1-19 protein from *P. falciparum (3D7)* (provided by Dr. Anthony Holder, Francis Crick Institute) were biotinylated and tetramerized as described above. Decoy reagent to gate out non-MSP1^+^ B cells was made by conjugating SA-PE to AF647 using an AF647 protein labeling kit (ThermoFisher), washing and removing any unbound AF647, and incubating with an excess of an irrelevant biotinylated HIS-tagged protein, similar to what has been previously described (Taylor et al., 2012a).

### Mouse and Human Cell Enrichment and Flow Cytometry

For murine samples, splenic cell suspensions were prepared and resuspended in 200 μl in PBS containing 2% FBS and Fc block (2.4G2) and first incubated with Decoy tetramer at a concentration of 10 nM at room temperature for 10 min. MSP1-PE tetramer was added at a concentration of 10 nM and incubated on ice for 30 min. Cells were washed, incubated with anti-PE magnetic beads for 30 min on ice and passed over magnetized LS columns (Miltenyi Biotec) to elute the bound cells as previously described (Taylor et al., 2012a). For human samples, PBMC were similarly stained and enriched using Decoy, PfAMA1, and PfMSP1 tetramers. All bound cells were stained with surface antibodies followed by intracellular antibody staining when needed (Table S1). All cells were run on the LSRII (BD) and analyzed using FlowJo software (Treestar).

### Single Cell BCR Sequencing and Cloning

Single MSP1^+^ MBCs were FACS sorted using an ARIAII into 96-well plates. BCRs were amplified and sequenced from the cDNA of single cells as previously described (Schwartz et al., 2014), with additional IgH primers used (Tiller et al., 2009). Amplified products were cloned and generated mAbs using previously described methods (Schwartz et al., 2014, Tiller et al., 2009).

### ELISAs

Costar 96-well EIA/RIA plates (Fisher Scientific) were coated overnight at 4°C with 10 μg/ml of MSP1 protein. Plates were blocked with 2% BSA prior to sample incubation. For serum samples, plates were incubated with serially diluted serum from naive or infected animals. For cloned mAbs, plates were incubated with serially diluted mAbs starting at 10 ng/μl. Each sample was plated in duplicate. For serum samples, bound antibodies were detected using either IgM Biotin (II/41), IgG Biotin (Poly4053), IgG1 Biotin (A85-1), IgG2c Biotin (5.7), IgG2b Biotin (R12-3), or IgG3 (R40-82) followed by Streptavidin-HRP (BD). For mAbs, bound antibodies were detected with mouse anti-human IgG-HRP (SouthernBiotech). Absorbance was measured at 450 nm using an iMark Microplate Reader (Bio-Rad).

### ELISPOT

96-well ELISPOT plates (Millipore) were coated overnight at 4°C with 10 μg/ml of Ig(H+L) unlabeled antibody (Southern Biotech). Plates were blocked with 10% FBS in complete DMEM (GIBCO). MSP1^+^ MBCs were sorted using a FACSAria (BD) from memory mice 2 days after rechallenge. Cells of each MBC population were plated onto coated ELISPOT plates and incubated at 37°C for an additional 2.5 days. Cells were washed off and secreted antibodies were detected using either IgM Biotin (II/41) or IgG Biotin (Poly4053) followed by Streptavidin-HRP (BD). Nonspecific (background) spots were determined in wells containing no cells. Spots were developed using AEC substrate (BD) and counted and analyzed using the CTL ELISPOT reader and Immunospot analysis software (Cellular Technology Limited). Number of spots detected per well were used to calculate spot frequency per 1 × 10^5^ total cells.

### Depletion of CD4+ T Cells

For depletion of CD4^+^ T cells, GK1.5 monoclonal antibody to CD4 (rIgG2b; BioXcell) was used. One and two days prior to secondary challenge, memory mice were given an i.p. injection of 200 μg GK1.5 or isotype control diluted in PBS. Efficiency of CD4^+^ T cell depletion was monitored by checking blood of mice pre-depletion, day 1 post injection and day of challenge. Depletion was found to be greater than 98% of CD4^+^ T cells as assessed by a non-GK1.5 competing anti-CD4 clone, RM4-4.

### Statistical Analysis

Unpaired, two-tailed Student’s t tests were applied to determine the statistical significance of the differences between groups with Prism (Graphpad) software. The p values were considered significant when p < 0.05 (^∗^), p < 0.01 (^∗∗^), and p < 0.001 (^∗∗∗^).

## Author Contributions

A.T.K. designed and performed experiments, analyzed data, and wrote the manuscript. C.D.T. performed sequencing analysis and cloning. G.J.K. and K.S.K. performed experiments and helped with manuscript preparation. A.H. provided *P. falciparum* MSP1 protein and reviewed manuscript. S.P. and P.D.C. provided human samples, expertise with human experiments, and reviewed the manuscript. D.J.R. designed sequencing and cloning experiments and reviewed manuscript. M.P. designed experiments, analyzed data, and wrote the manuscript.

## Supplemental Information

Document S1. Figures S1–S6, Table S1, and Supplemental Experimental Procedures

Document S2. Article plus Supplemental Information
