# Supplementary material for: Somatically Hypermutated Plasmodium-Specific IgM+ Memory B Cells Are Rapid, Plastic, Early Responders upon Malaria Rechallenge
Source: Immunity. 2016 Aug 16;45(2):402–14. doi: 10.1016/j.immuni.2016.06.014 (PMC5118370; doi:10.1016/j.immuni.2016.06.014)
Supplement: Document S1. Figures S1–S6, Table S1, and Supplemental Experimental Procedures [file mmc1.pdf]

Immunity, Volume *45*

Supplemental Information

Somatically Hypermutated *Plasmodium*-Specific IgM$^+$ Memory B Cells Are Rapid, Plastic, Early Responders upon Malaria Rechallenge

Akshay T. Krishnamurty, Christopher D. Thouvenel, Silvia Portugal, Gladys J. Keitany, Karen S. Kim, Anthony Holder, Peter D. Crompton, David J. Rawlings, and Marion Pepper

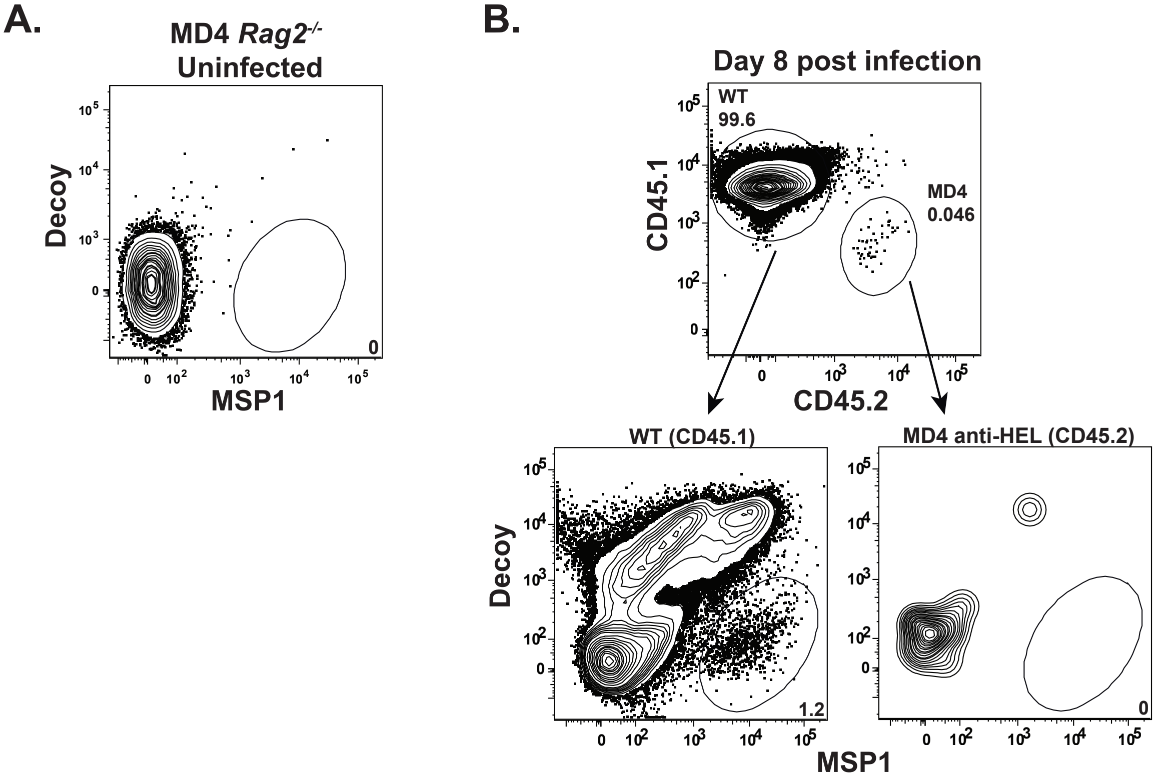

Figure S1 related to Figure 1. MSP1-specific B cells bind tetramer and expand in an antigen specific manner. (A) Representative plots to identify $MSP1^+$ B cells from spleens of naïve MD4-*Rag2*$^{-/-}$ mice. (B) $CD45.2^+$ MD4-*Rag*$^{-/-}$ splenocytes were transferred into CD45.1 WT B6 mice and infected with $1x10^6$ iRBCs the following day. 8 days post infection recipient mice spleens were enriched with Decoy and MSP1 B cell tetramers to identify $MSP1^+$ B cells from either CD45.1 WT recipient cells or donor $CD45.2^+$ MD4-*Rag*$^{-/-}$ cells.

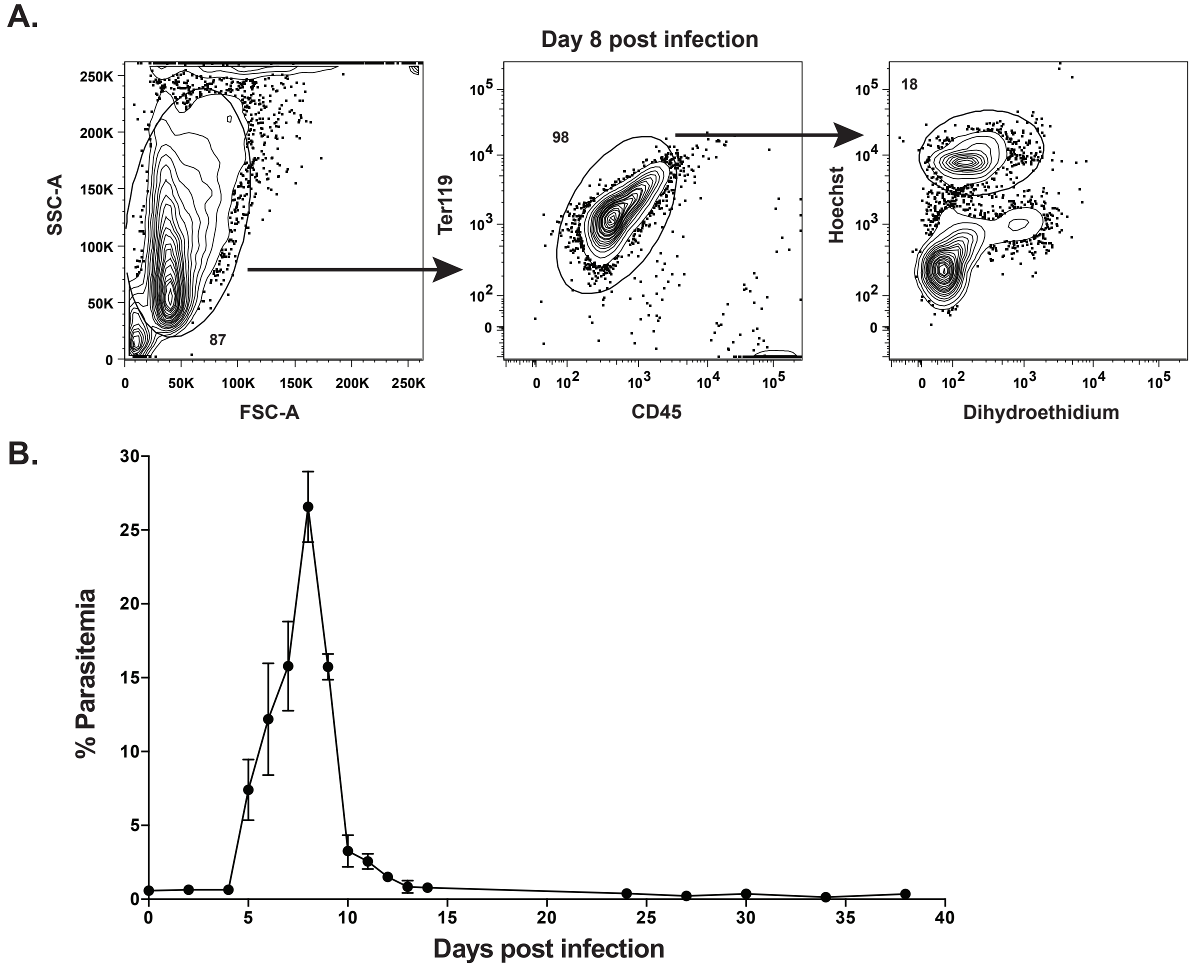

Figure S2 related to Figure 1 and Figure 2. Measurement of parasitemia by flow cytometry. (A) Representative gating scheme to identify *P.chabaudi* iRBCs gated as $Ter119^{+}CD45^{-}Hoechst^{+}$ in 1ul of blood from a WT mouse 8 days post infection. (B) Course of parasitemia in WT mice measured by flow cytometry after infection with $1x10^{6}$ iRBCs over the course of 40 days. Each data point shows mean ± SEM with 3-8 mice per timepoint from at least two independent experiments.

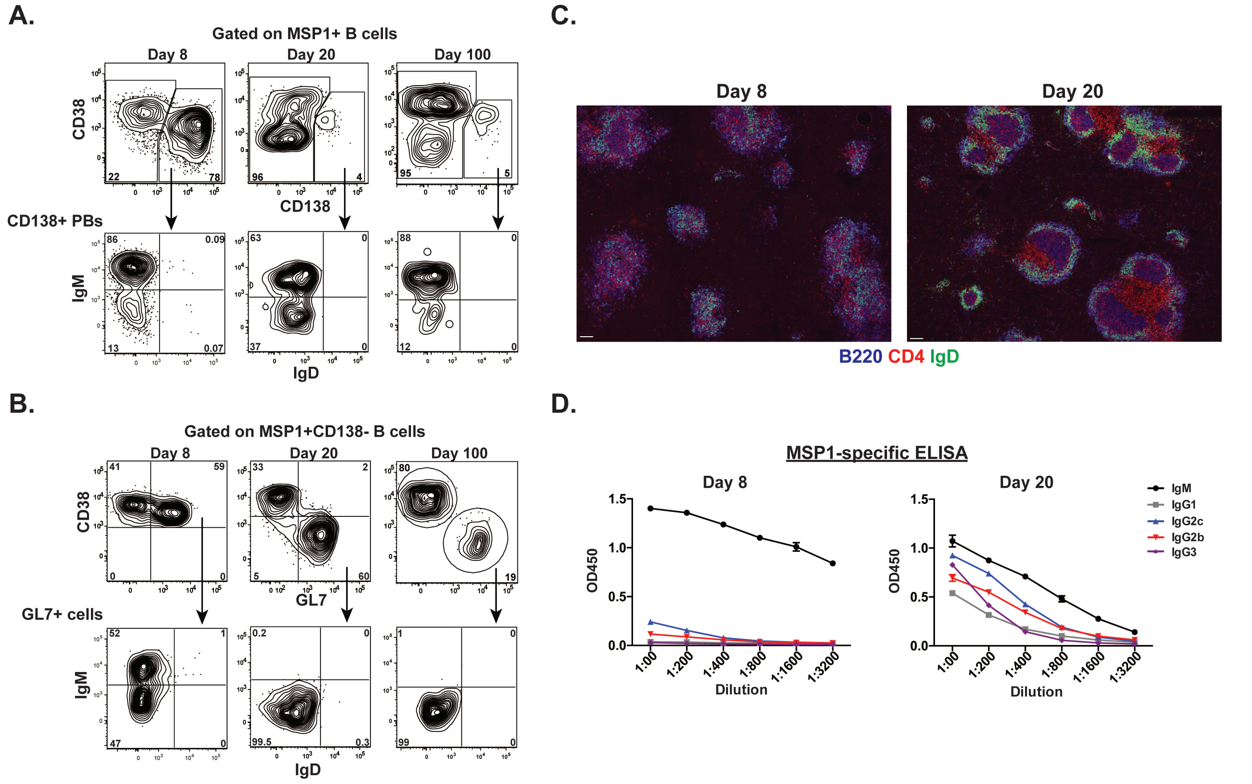

Figure S3 related to Figure 2. Splenic histology and serum antibody analysis reflect cellular kinetics during early, acute phase of infection. (A) Representative plots of CD38 and CD138 expression (top row) and IgM and IgD expression (bottom row) on CD138$^+$ cells of MSP1$^+$ B cells on days 8, 20, and 100 post infection with 1x10^6 *Pc* iRBCs. (B) Representative plots of CD38 and GL7 expression (top row) and IgM and IgD expression (bottom row) on GL7$^+$ cells of MSP1$^+$CD138$^-$ cells on days 8, 20, and 100 post infection with 1x10^6 *Pc* iRBCs. (C) Representative splenic sections from mice 8 or 20 days post infection with 1x10^6 *Pc* iRBCs stained with CD4 (red), B220 (blue), and IgD (green). Composite pictures generated by stitching together multiple 10x images over large area of spleen. Scale bars, 100µm. (D) Serum antibody analysis by ELISA for MSP1-specific IgM, IgG1, IgG2b, IgG2c, and IgG3 from individual mice on days 8 and 20 post infection with 1x10^6 *Pc* iRBCs. Each dilution point shows mean ± SEM. Graphs represent combined data from 3 independent experiments with 3 mice per group.

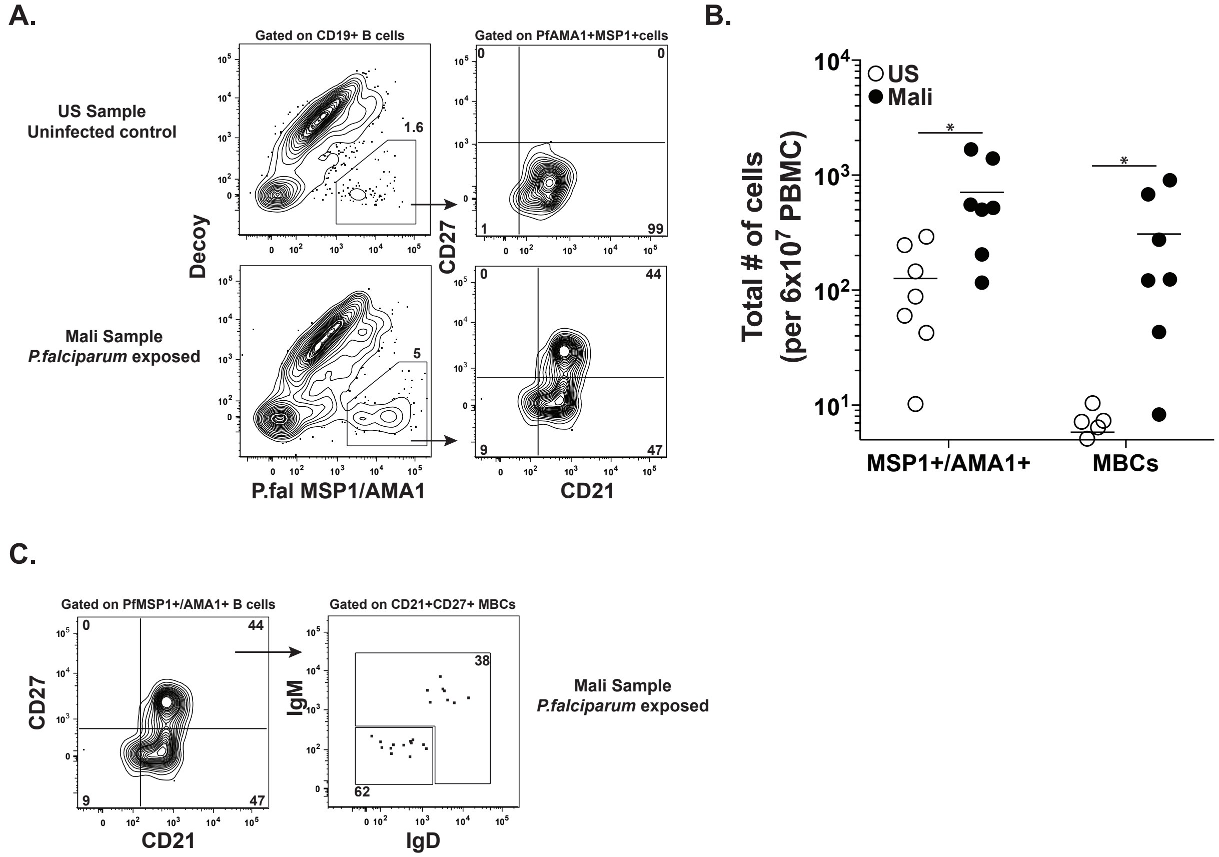

Figure S4 related to Figure 3. Human *Plasmodium*-specific MBCs are phenotypically heterogeneous. $CD19^+$ human B cells identified in US and Mali PBMC after excluding $CD3^+CD14^+CD16^+$ non-B cells and enrichment with PfMSP1, PfAMA1 and Decoy tetramers. (A) Representative plots of $PfMSP1/AMA1^+$ B cells and CD21/CD27 expression. (B) Total $PfMSP1/AMA1^+$ B cells and MBCs per $6x10^7$ PBMC in US and Mali PBMC. Data is combined from 3 independent experiments with 7 samples per group. Line indicates mean. *$p<0.05$ (C) Representative plot of IgM and IgD expression on $CD27^+CD21^+$ memory B cells from Mali sample.

A.

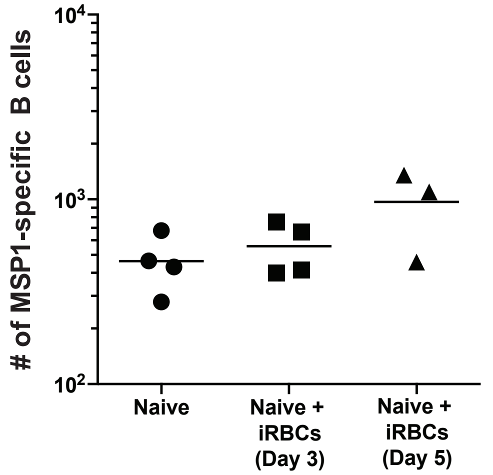

B.

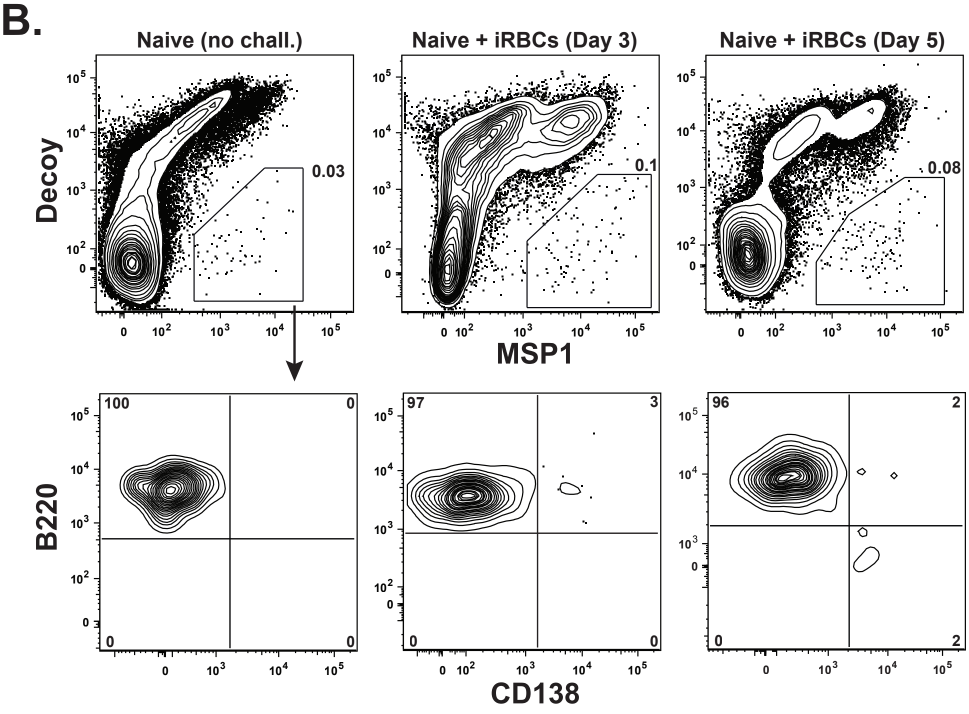

Figure S5 related to Figure 4. Naïve MSP1$^+$ B cells do not differentiate or secrete antibody after primary infection with 1x10^7 iRBCs. (A) Representative plots identifying MSP1$^+$ B cells (top row) and B220 and CD138 expression (bottom row) in uninfected naïve mice or naïve mice 3 or 5 days post primary infection with 1x10^7 iRBCs injected i.v. (B) Total number of MSP1$^+$ B cells in uninfected naïve mice or naïve mice 3 or 5 days post primary infection with 1x10^7 iRBCs injected i.v. Data is combined from 2 independent experiments with 3-5 mice per group.

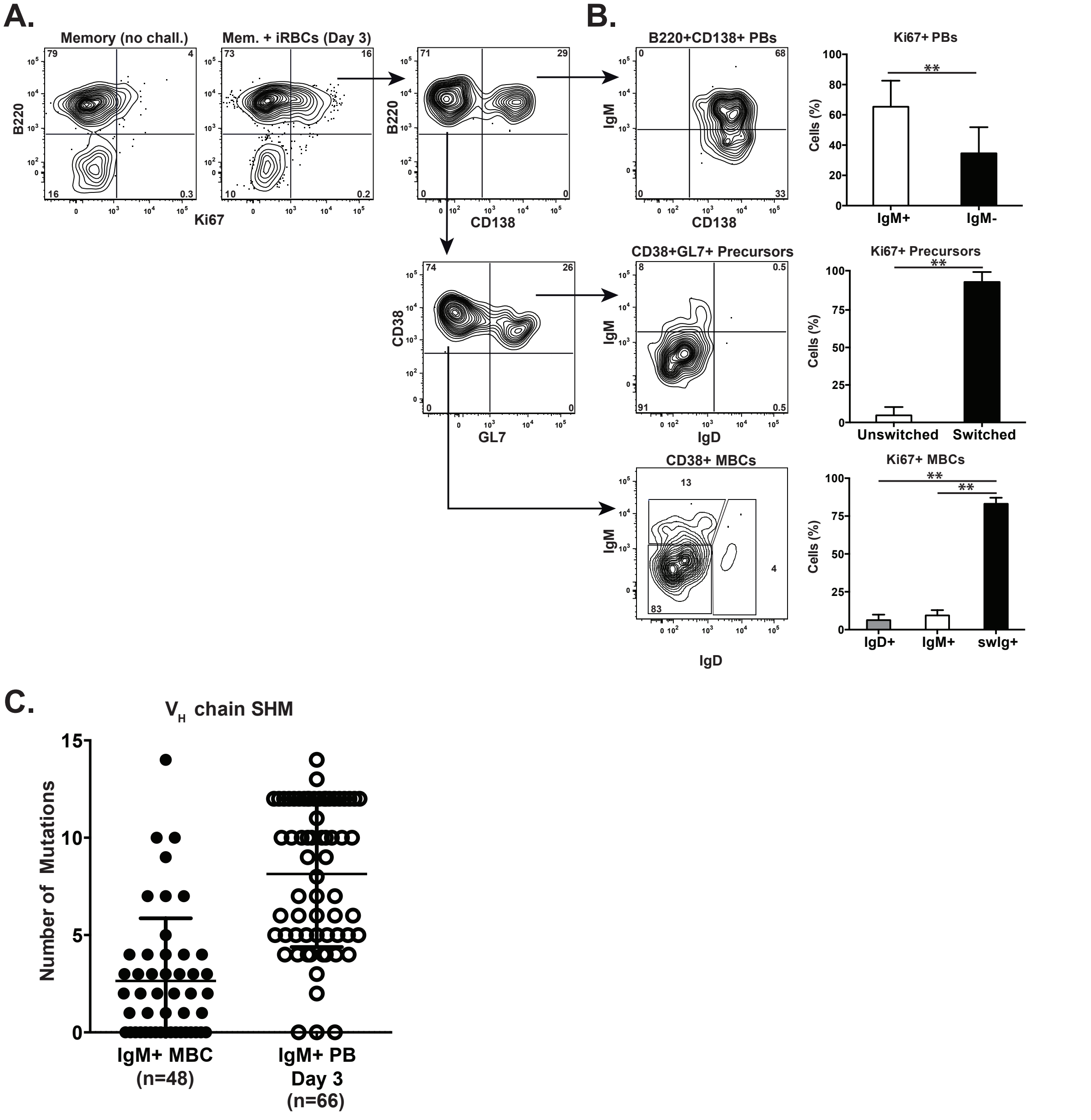

Figure S6 related to Figure 5. Phenotype of newly formed proliferating MSP1-specific B cells after a secondary infection. (A) Columns 1 and 2: Ki67 expression on B220$^{+/-}$ MSP1^{+} B cells in memory mice pre or post iRBC challenge (day 3). Column 3: representative plots of B220 and CD138 expression (top) and CD38 and GL7 expression (middle) of Ki67^{+} cells in rechallenged memory mice. (B) Representative plots of IgM and IgD expression on Ki67^{+}B220^{+} PBs (top), precursors (middle), and MBCs (bottom). Adjacent graph shows percentage of indicated isotypes of each population. Bar graphs represents data combined from 2 independent experiments with 6 mice per group. Error bars show SD. **p<0.01 (C) Number of mutations in the heavy chain (V_H) of individual MSP1^{+} IgM^{+} MBCs 100 days p.i. prior to challenge and MSP1^{+}IgM^{+}B220^{+}CD138^{+} plasmablasts from memory mice 3 days after challenge with 1x10^{7} iRBCs. Each dot indicates a single cell. Line indicates mean. Data combined from 3 independent experiments.

Specificity	Antibody	Clone	Color
Mouse	B220	RA3-6B2	BV711, APC eFluor 780
Mouse	IgM	II/41	APC, BV786
Mouse	IgD	11-26c.2a	BV650
Mouse	CD3	145-2C11	PerCPCy5.5
Mouse	F4/80	BM8	PerCpCy5.5
Mouse	CD38	90	Alexa Fluor 700
Mouse	CD138	281-2	BV605
Mouse	GL7	GL7	eFluor450
Mouse	CD73	Ty-23	PE-Cy7
Mouse	CD80	16-10A1	FITC
Mouse	CD45.1	A20	APC eFluor 780
Mouse	CD45.2	104	APC
Mouse	Ki67 (ICS)	SolA15	FITC, APC
Mouse	Ig(H+L) (ICS)		Alexa Flour 350
Human	CD19	HIB19	Alexa Flour 700
Human	CD20	2H7	PerCpCy5.5
Human	CD3	UCHT1	BV711
Human	CD14	MOP9	BV711
Human	CD16	3G8	BV711
Human	IgM	MHM-88	BV510
Human	IgD	IA6-2	PE-Cy7
Human	CD27	0323	APC-Cy7
Human	CD21	BL13	FITC

Table S1 related to Experimental Procedures. Mouse and human surface and intracellular antibodies for staining Plasmodium-specific B cells. For murine and human samples, the following surface antibodies were used in various combinations (purchased from BD Biosciences, Ebioscience, or Biolegend). For intracellular staining (ICS), cells were fixed and permed with BD Cytofix/Cytoperm and washed and stained in BD perm buffer.

Supplemental Experimental Procedures

Parasitemia by flow cytometry

Parasitemia was measured by flow cytometry by staining 1ul of blood with Ter119 APC eFluor780 (eBioscience), CD45 APC (BD), Hoechst33342 (Sigma), and Dihydroethidium (Sigma). Giemsa staining of thin blood smears was done in parallel.

Human PBMC samples

Deidentified *Plasmodium*-infected PBMC samples are from a previously described cohort in Mali previously described. (Crompton et al., 2008). Uninfected control PBMC are from from healthy U.S. adult donors enrolled in NIH protocol #99-CC-0168. Demographic and travel history data were not available from the anonymous U.S. donors, but prior *P.falciparum* exposure is unlikely.

Immunofluorescence staining of spleens

Spleens from infected mice were embedded in OCT and flash frozen. 8um sections were cut and fixed in acetone and then stained with CD4 Biotin (RM4-5), B220 Alexa Fluor 647 (RA3-6B2), and IgD Alexa Fluor 488 (11-26c.2a). Streptavidin Cy3 (Jackson Immunoresearch) was used as a secondary antibody. Images were acquired using a Nikon Eclipse 90i microscope and NIS Elements BR (Build 738) software was used for the capture of individual images for each channel. Raw TIFF files were imported in Adobe Photoshop for overlay of single channel images and editing.
